# Proteomic analysis identifies deregulated metabolic and oxidative-associated proteins in Italian intrahepatic cholangiocarcinoma patients

**DOI:** 10.1186/s12885-021-08576-z

**Published:** 2021-07-28

**Authors:** Giuliana Cavalloni, Caterina Peraldo-Neia, Annamaria Massa, Carlo Bergamini, Alessandro Trentini, Giovanni De Rosa, Lorenzo Daniele, Fabiola Ciccosanti, Carlo Cervellati, Francesco Leone, Massimo Aglietta

**Affiliations:** 1 Division of Medical Oncology, Candiolo Cancer Institute, FPO-IRCCS, Candiolo, Torino, Italy; 2 Cancer Genomics Lab, Fondazione Edo ed Elvo Tempia, Biella, Italy; 3 Department of Chemistry and Pharmaceutical Sciences, University of Ferrara, Ferrara, Italy; 4 Pathology Unit, AO Ordine Mauriziano, Torino, Italy; 5 Department of Epidemiology, Preclinical Research, and Advanced Diagnostics, National Institute for Infectious Diseases, IRCCS ‘Lazzaro Spallanzani’, Rome, Italy; 6 Department of Morphology, Surgery & Experimental Medicine, University of Ferrara, Ferrara, Italy; 7 Department of Oncology, ASL BI, Ospedale degli Infermi di Biella, Ponderano, BI Italy; 8 Department of Oncology, University of Turin, Torino, Italy

**Keywords:** Intrahepatic cholangiocarcinoma, Proteomics, Mass spectrometry, Metabolism, ROS

## Abstract

**Background:**

Cholangiocarcinoma (CCA) is an aggressive disease with poor prognosis. A molecular classification based on mutational, methylation and transcriptomic features could allow identifying tailored therapies to improve CCA patient outcome. Proteomic remains partially unexplored; here, we analyzed the proteomic profile of five intrahepatic cholangiocarcinoma (ICC) derived from Italian patients undergone surgery and one normal bile duct cell line.

**Methods:**

Proteome profile was investigated by using 2D electrophoresis followed by Mass Spectrometry (MS). To validate proteomic data, the expression of four overexpressed proteins (CAT, SOD, PRDX6, DBI/ACBP) was evaluated by immunohistochemistry in an independent cohort of formalin fixed, paraffin-embedded (FFPE) ICC tissues. We also compared proteomic data with those obtained by transcriptomic profile evaluated by microarray analysis of the same tissues.

**Results:**

We identified 19 differentially expressed protein spots, which were further characterized by MS; 13 of them were up- and 6 were down-regulated in ICC. These proteins are mainly involved in redox processes (CAT, SODM, PRDX2, PRDX6), in metabolism (ACBP, ACY1, UCRI, FTCD, HCMS2), and cell structure and organization (TUB2, ACTB). CAT is overexpressed in 86% of patients, PRDX6 in 73%, SODM in 100%, and DBI/ACBP in 81% compared to normal adjacent tissues. A concordance of 50% between proteomic and transcriptomic data was observed.

**Conclusions:**

This study pointed out that the impairment of the metabolic and antioxidant systems, with a subsequent accumulation of free radicals, might be a key step in CCA development and progression.

**Supplementary Information:**

The online version contains supplementary material available at 10.1186/s12885-021-08576-z.

## Background

Cholangiocarcinoma (CCA) is the second most common type of hepatobiliary cancer arising from the ductal epithelium of the biliary tree, either within the liver (intrahepatic cholangiocarcinoma, ICC) or from extrahepatic bile ducts (extrahepatic cholangiocarcinoma, ECC), which included perihilar and distal cholangiocarcinoma [1]. CCA incidence, pathogenesis and etiology differ not only among the subtypes, but also according to ethnicity, with peculiar genetic alterations and risk factors [2]. Globally, its incidence is higher in Asiatic Countries of the Pacific area, especially due to parasite infection, but the number of cases in Europe is increasing in the last 30 years [3]. As it concerns Italy, a retrospective study demonstrated that CCA incidence in the years 1988–2005 displayed an annual increment of 3–6%, with highly increased rate and mortality for ICC compared to ECC [4]. A minority of patients  had surgically resectable tumors at diagnosis, but the recurrence rate was higher than 50% within 5 years, since the diagnosis was often delayed [5]. The therapeutic approach for locally advanced or metastatic diseases is chemotherapy; the backbone is represented by Gemcitabine in association with platinum compounds, with a median overall survival of 11.7 months compared to 8.1 months of gemcitabine alone [6]. Unfortunately, patients developed resistance and disease progression occurred, making this pathology highly lethal. The molecular mechanisms and genetic steps underlying the pathogenesis of this tumor remain largely unknown; the heterogeneity of these tumors, the different etiology and risk factors involved in tumor development, complicate the identification of suitable molecular target and treatment options. In the last years, the importance to properly classify CCA emerged, considering the subtypes as different entities. Large-scale technologies, such as whole genome sequencing, RNA-seq, microarray and methylation arrays, highlighted the real need to distinguish either the subtypes or the intra- and inter-tumoral heterogeneity of CCA [7–9]. It is well-known that some mutations such as *IDH1, BAP1, ARID1A,* and *FGFR2* rearrangements are typically enriched in ICC, while *KRAS* and *TP53* in ECC [10]. In general, *IDH1* and *FGFR2* aberrations are associated with better prognosis, while *KRAS* and *TP53* with worse outcome [11, 12]. These data enforced the real need to treat ICC and ECC with tailored clinical approaches. Indeed, not only mutations, but also distinct patterns of epigenetic alteration profiling may differentiate ICC from ECC [13]. Moreover, recent studies demonstrated that a classification based on etiology and molecular aspects, such as methylation and copy number variations, is complementary and more useful than the subtypes classification alone [14].

Although there are lots of information about transcriptomic and mutational status, the proteomic profile of CCA remains only partially explored and is mainly associated to particular histotypes and/or ethnic origin, in turn strictly associated with risk factors.

In a study of Dos Santos and coll. a panel of 39 altered proteins involved in motility and actin cytoskeleton remodeling was found in an ICC case series compared to non-tumoral adjacent liver tissue [15]. In an Asiatic case series, Annexin A2, peroxiredoxin 1 and, ezrin-radixin-moesin–binding phosphoprotein 50 were identified as negative prognostic markers of CCA patients [16, 17]. Kristiansen and coll analyzed a case series of CCA, identifying some deregulated proteins, some of them never been associated with CCA arising and progression [18].

A recent review summarized the uniqueness molecular profile of liver fluke-associated CCA obtained by combining multi-omics approaches. Anti-inflammatory, immunomodulator/immunosuppressor, epidermal growth factor receptor or platelet-derived growth factor receptor inhibitors, multi-targeted tyrosine kinase inhibitor, IL6 antagonist, Nuclear Factor-κB inhibitor, histone modulator, proteasome inhibitor MetAP2 inhibitor, 1,25(OH)_2_D_3_ and cyclosporine A are suggested as targets for the treatment of this tumor subtype [19].

Recently, the comparison of 6 tumor and peritumoral ECC tissues identified 233 de-regulated proteins, one of them, the up-regulated PPP3CA, is a strong predictor of poor survival [20].

To date, no proteome profiling has been explored in CCA derived from Italian patients. Here, we selected a homogeneous series of five ICC tumors obtained at the time of surgery. We processed them with 2-dimensional (2D) electrophoresis followed by mass spectrometry. The comparison of the proteomic profile with that obtained from a normal epithelial bile duct cell line provided precious information about the pathways potentially involved in tumor development and progression.

## Methods

### Cell line and tumor samples

Normal biliary epithelium cell line HIBEpiC (ScienCell Research Laboratories, Carlsbad, CA) was cultured in RPMI 1640 (Gibco, Carlsbad, CA, USA) medium supplemented with 10% fetal bovine serum (Sigma–Aldrich, St. Louis,MO, USA) and 1% penicillin/streptomycin (Life Technologies Gathersburg, MD) at 37 °C and 5% CO2. Tumor samples, 5 ICC fresh frozen (FF) tissues and 15 ICC formalin fixed, paraffin embedded (FFPE), were collected from ICC patients of Italian origin. Among FF ICC samples, 3 derived from females, 2 from males, with a median age at the time of diagnosis of 69 years. Tumor tissues used for the experiments were macrodissected from surgical samples, avoiding the inner (more necrotic) and the peripheral part of tumors (useful for margins evaluation in diagnosis). Biological material was obtained after informed consent, following institutional review board-approved protocols (001-IRCC-00IIS-10 FPO-IRCCS, Istituto di Ricovero e Cura a Carattere Scientifico Candiolo (TO), Italy).

### Proteomic analysis by 2 dimensional (2D) electrophoresis and mass spectrometry

Five ICC and one normal epithelial bile duct cell line were subjected to proteomic analysis. Cells in culture were harvested and centrifuged at 400 g for 10 min at room temperature and washed once in 0.3 M sucrose. The pellet was collected and treated with 100 μl/1 × 10^6^ cells of lysis buffer (7 M Urea, 2 M Thiourea, 4% CHAPS, 1 mM EDTA, 2 mM PMSF, 1 mM NaF, 40 mM Tris, pH 9) containing protease inhibitors (Halt Protease, Thermo Fisher Scientific, Cat. No. 78429). After vortexing 3 times for 10 s, the cells were sonicated at 20 kHz and 4 °C for pulses of 20 s with 20 s rest, for a total processing time of 3 min. The suspension was incubated for 30 min on a rotating wheel and then centrifuged 30 min at 13000 xg and 4 °C to remove particulate material. The supernatant was collected, placed in a clean microcentrifuge tube and centrifuged again 15 min at 13000 xg and 4 °C. At the end of the procedure, the supernatant was collected in a clean microcentrifuge tube and total protein content was determined with the Bradford assay.

For tissue samples, the procedure was slightly different. Samples were washed twice with sterile normal saline (0.15 M NaCl) under a laminar flow hood to remove contaminating hemoglobin and minced with a sterile scalpel. The small pieces were then transferred in a tube together with lysis buffer plus protease inhibitors in a weight (g) to volume (ml) ratio of 1:3. The samples were then subjected to homogenization by using a Polytron Homogenizer with brief cycles and on ice bath. At the end of the procedure, samples were transferred in clean microcentrifuge tubes and centrifuged for 30 min at 13000 xg and 4 °C. The supernatants were collected and subjected to another centrifugation at 15 min at 13000 xg and 4 °C. Finally, supernatants were collected and assayed for total protein content with the Bradford assay.

All samples were processed separately and subjected to 2D electrophoresis as follow. Tree hundreds μg of proteins were subjected to isoelectric focusing (IEF) and separated on 2D SDS-PAGE on 12.5% polyacrylamide gels as described by Carcoforo and collaborators [21]. Gels were stained with colloidal Coomassie, and scanned with the Molecular Imager PharosFX System. The analysis was then performed using the ProteomWeaver 4 program (Bio-Rad, Hercules, CA, USA). Protein spots were automatically identified and manually adjusted if needed, then merged by Pair Matching or Multi Matching function in the software. Each spot was normalized by the total density of the gel to account for possible differences in stain procedure and amount of protein loaded. Differences in spot intensities between ICC and control were considered significant if the matched spots had a fold change > 2 for the upregulated and < 0.5 for downregulated signals and a *p*-value < 0.01 in Student’s t-test. Differentially expressed spots were then processed for mass spectrometry-based peptide identification.

Briefly, gel fragments were washed in 100 mM ammonium bicarbonate and 50% (v/v) ACN, dehydrated by incubation in 100% (v/v) ACN and rehydrated in 50 mM ammonium bicarbonate containing 4 ng/μL of trypsin; 50 mM ammonium bicarbonate was added following digestion overnight at 37 °C. Tryptic peptides were concentrated with ZipTip mC18 pipette tips (Millipore) and co-eluted onto the MALDI target in 1 μL of α-cyano-4-hydroxycinnamic acid matrix (5 mg/mL in 50% ACN, 0.1% TFA). MALDI-MS and MALDI-MS/MS were carried out with a 5800 MALDI TOF/TOF Analyzer (Sciex, Ontario—Canada) essentially as described by Carcoforo and collaborators [21] and detailed in supplementary methods (Additional file 1).

### Immunohistochemistry

Catalase expression was evaluated in 15 ICC tumors. Briefly, tumor sections were deparaffinized and rehydrated with graded of ethanol. The epitope retrieval was obtained using Antigen Retrieval Citrate solution pH 6.0 and exposed to 2 min cycles at 700 W in a microwave oven. Endogenous peroxidase activity was blocked with 0.3% hydrogen peroxide for 10 min, followed by treatment with V-block for 30 min (Dako, Santa Clara, CA). Sections were incubated for 2 h in a moist chamber at room temperature with the specific primary antibody for CAT (mouse anti Human CAT, 1:100, Santa Cruz) diluted in TBS 1X. Then, slides were rinsed twice in buffer and then incubated with the detection system solution, a Dextran polymer conjugated to horseradish peroxidase, for 30 min. The final reaction was visualized using 3,3′-diaminobenzidine in a buffer/hydrogen peroxide solution for 3 min. Finally, sections were counterstained with Harris’s hematoxylin, dehydrated, and mounted in DPX (Sigma Aldrich, Saint Louis, MO, USA). Eleven ICC tumors (for five samples, the biological material is not available and one more tumor tissue was added) were stained for PRDX6, SODM, and DBI (Aurogene). Briefly, after rehydration, the endogenous peroxidases were blocked in a solution of methanol and hydrogen peroxides (0.3%) for 30 min. The epitope retrieval was obtained using Antigen Retrieval Citrate solution pH 6.0 (Dako) and exposed to 2 cycles of 5 min each at 850 W in a microwave oven. The saturation of non-specific sites was performed with a solution of 5% Normal Goat Serum (Dako) in TBS-Tween (0.3%)-Triton (0.1%) for 1 h in moist chamber at room temperature. Then, slides were incubated O/N at 4 °C with the appropriate primary antibodies at the following dilutions: 1:50 for rabbit polyclonal PRDX6, 1:200 for mouse monoclonal SODM and rabbit polyclonal DBI in the saturation solution. After rinsing, slides were incubated with anti-Rabbit-HRP (for DBI and PRDX6) and with anti-Mouse-HRP (for SODM) for 1 h at room temperature. The final reaction was visualized using DAB+ Substrate Chromogen System (Dako) for 3 min. Finally, sections were counterstained with Harris’s hematoxylin, dehydrated, and mounted in DPX (Sigma Aldrich). Immunohistochemical results were evaluated by two different pathologists (LD and GDR). For CAT and ACBP/DBI expression, the intensity of the reaction was classified using a three grade system: weak positivity (a weak intensity cytoplasmatic staining observed in < 30% of the cells), intermediate positivity (a moderate intensity cytoplasmatic staining observed in > 30% of the tumor cells), and strong positivity (a strong intensity cytoplasmatic staining observed in > 30% of the tumor cells). For SODM the percentage of positive tumor cells was evaluated on a scale of 0–3 (0 no staining, 1+ < 10%, 2+ 11–30%, 3+ 31–50%, 4+ > 50%). For PRDX6 the staining was scored as the product of the staining intensity (on a scale of 0–2: negative = 0, low = 1, high = 2) and the percentage of cells stained (on a scale of 0–3:0 = zero, 1 = 1–25%, 2 = 26–50%, 3 = 51–100%) resulting in scores on a scale of 0–5.

### External dataset for gene expression profiling

We extrapolated the gene expression data of the 5 ICC tumors used for proteomic analysis from the dataset GSE107102. They were included in a bigger cohort of ICC tumors analyzed in our previous work [22]. Material and methods used are previously deeply described in Peraldo-Neia et al. [22]. Gene Expression Profiling Interactive analysis GEPIA (http://gepia.cancer-pku.cn) database and the Human Protein Atlas (https://www.proteinatlas.org) database were used to verify the expression at mRNA and protein levels, respectively, of differentially expressed targets selected by proteomic analysis.

## Results

### Analysis of differentially expressed proteins

Five tissue samples of ICC patients and normal control (normal biliary epithelium cell line) were run on 2-D GE to investigate differentially expressed protein in tumor compared to normal tissue. Approximately 580 spots were detected in 2-D GE, as shown in Table 1, with an average of 218 spots/gel.

**Table 1:** Number of spots detected for each sample and control cells

Sample	Number of spots
Control cells	233
Sample 1	224
Sample 2	197
Sample 3	227
Sample 4	218
Sample 5	212

**Fig. 1:**
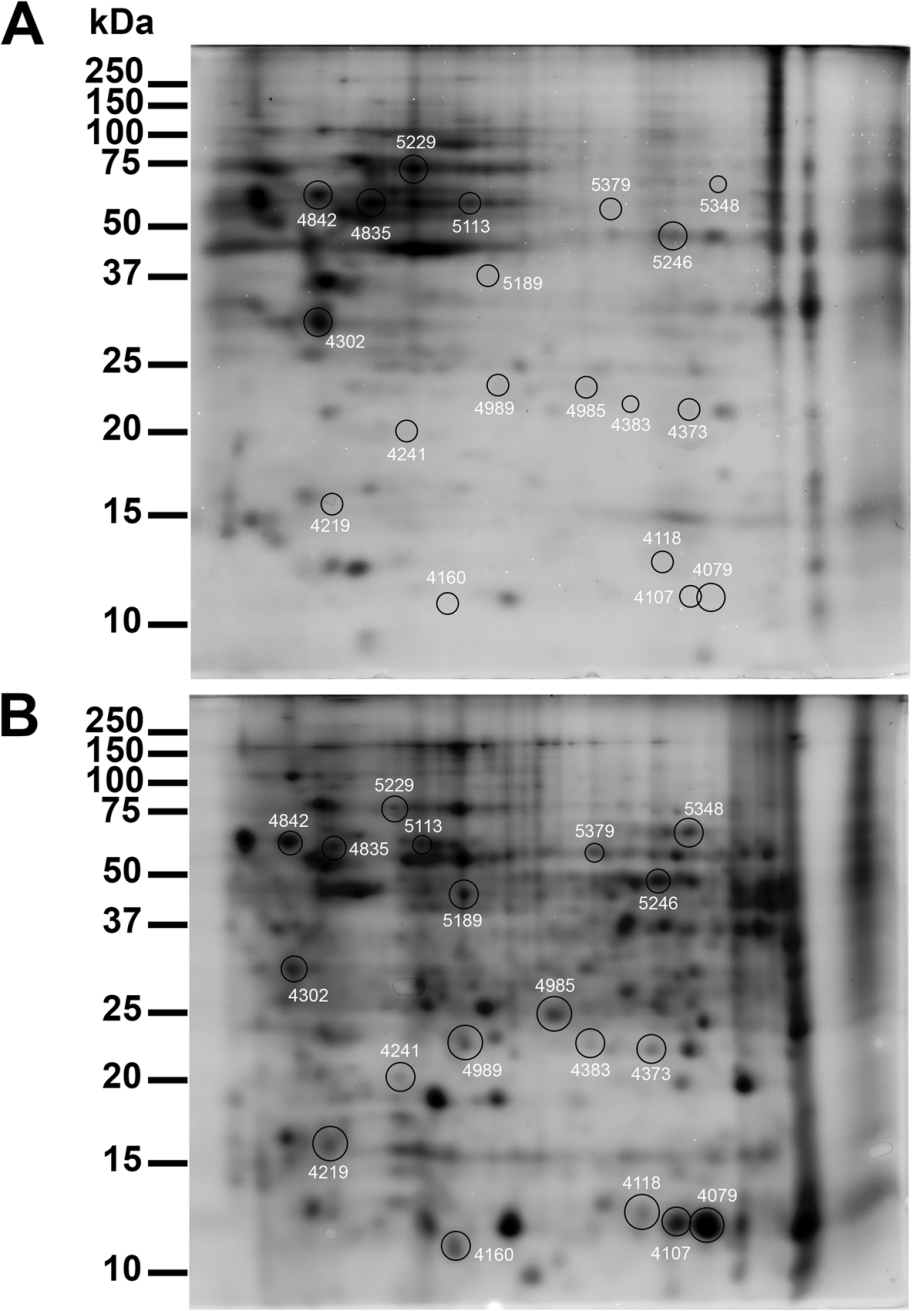
Representative 2D gel images indicating the differential spots in normal cells (**A**) and ICC samples (**B**). Black circles indicate up-regulated spots. Immobilized pH gradient 3–10 NL strips were used for the first dimension, 12.5% polyacrylamide gels were used for the second dimension

Proteins of each sample were run on separate gels. Figure 1 showed a representative image of gels for one sample (A) and for the normal bile duct cell line (B), while the images for the other samples run in different gels are collected in Additional file 2.

The analysis indicated that 19 proteins, (one of them, HBB, was identified by two peptides) were differentially expressed within the two groups. In particular, 13 spots were upregulated (> 2 fold) and 6 were downregulated (< 0.5 fold) in ICC samples.

Mass Spectrometry was used to identify the selected 19 protein spots, which is reported in Table 2.

**Table 2:** Up- and down-regulated proteins identified by proteomic analysis and MS in ICC compared to control

Acc. n° UniProtKB	SPOT ID	Protein name	Pathway	Fold Change	Sequence coverage (%)	Number of Unique Peptides (C.I. 95%)
**Upregulated proteins**						
sp|P07108|ACBP_HUMAN	**4160**	Acyl-CoA-binding protein	Metabolism (lipids)	**14.3**	72.4	7
sp|P68871|HBB_HUMAN	**4079**	Hemoglobin subunit beta	Oxygen transport	**14.9**	89.8	18
sp|P68871|HBB_HUMAN	**4107**	Hemoglobin subunit beta	Oxygen transport	**14.4**	89.8	18
sp|P04040|CATA_HUMAN	**5348**	Catalase	Redox homeostasis	**10.6**	21.3	6
sp|Q03154|ACY1_HUMAN	**5189**	Aminoacylase-1	Metabolism (amino acids)	**7.1**	47.3	11
sp|P47985|UCRI_HUMAN	**4383**	Cytochrome b-c1 complex subunit Rieske, mitochondrial	Metabolism	**5.6**	20.8	4
sp|P63261|ACTG_HUMAN	**4219**	Actin, cytoplasmic 2	Cytoskeleton	**4.8**	29.1	5
sp|P30041|PRDX6_HUMAN	**4985**	Peroxiredoxin-6	Redox homeostasis	**3.7**	64.7	10
sp|P32119|PRDX2_HUMAN	**4241**	Peroxiredoxin-2	Redox homeostasis	**3.7**	36.4	8
sp|Q96IU4|ABHEB_HUMAN	**4989**	Protein ABHD14B	Cytosolic sulfonation of small molecules	**3.7**	47.1	5
sp|P04179|SODM_HUMAN	**4373**	Superoxide dismutase [Mn], mitochondrial	Redox homeostasis	**3.5**	39.2	6
sp|P62805|H4_HUMAN	**4118**	Histone H4	DNA binding	**3.0**	17.5	2
sp|P00352|AL1A1_HUMAN	**5379**	Retinal dehydrogenase 1	Cell signaling	**3.0**	24	8
**Downregulated proteins**						
sp|P54868|HMCS2_HUMAN	**5246**	Hydroxymethylglutaryl-CoA synthase, mitochondrial	Metabolism	**0.45**	29.9	6
sp|P07237|PDIA1_HUMAN	**4842**	Protein disulfide-isomerase	Metabolism	**0.33**	32.5	9
sp|O95954|FTCD_HUMAN	**5113**	Formimidoyltransferase-cyclodeaminase	Metabolism	**0.29**	39.7	8
sp|P38646|GRP75_HUMAN	**5229**	Stress-70 protein, mitochondrial	Heat shock response	**0.23**	15.5	7
sp|P68363|TBA1B_HUMAN	**4835**	Tubulin alpha-1B chain	Cytoskeleton	**0.22**	26.8	7
sp|P06753|TPM3_HUMAN	**4302**	Tropomyosin alpha-3 chain	Cytoskeleton	**0.18**	27.4	5

Fold change represents the ratio between the mean percentage relative volume (%V) (%V = V(single spot)/V(total spot)) determined in ICC and the normal sample. Score refers to the sum of the Mascot ion scores of all of the peptides that were identified for a given protein. Sequence coverage is the % of aminoacidic sequences identified by MS. Number of Unique Peptides is the number of peptides matching the identified proteins

A consistent part of upregulated proteins (23.5%) in ICC tissues, is related to redox biology. In particular CAT, PRDX2, PRDX6 and SODM are highly expressed compared to normal biliary epithelium cell line (10.6, 3.7, 3.7 and 3.5 fold, respectively). Other proteins with an increased expression were related to metabolism (Acyl-CoA-binding protein, Aminoacylase-1, Cytochrome b-c1 complex subunit Rieske, mitochondrial), cell structure (cytoplasmic Actin 2), signaling (Retinal dehydrogenase 1), oxygen transport (Hemoglobin subunit beta) and DNA binding (Histone H4). Downregulated proteins (fold change between 0.45–0.18) are involved in cell metabolism (Hydroxymethylglutaryl-CoA synthase, mitochondrial Protein disulfide-isomerase and Formimidoyltransferase-cyclodeaminase), cytoskeleton organization (Tubulin alpha-1B chain and Tropomyosin alpha-3 chain), and heat shock protein (Stress-70 protein, mitochondrial).

### Immunohistochemistry validation of redox and metabolism processes

In order to validate proteomic data, we selected four among the up-regulated proteins found by previous analysis, all associated to overrepresented above mentioned processes; we evaluated CAT (*n* = 15), SODM, PRDX6 and DBI/ACBP (*n* = 11 for the last three proteins) protein expression by IHC in an independent case series of archival tissue samples derived from ICC Italian patients. Table 3 summarized the score staining for each protein.

**Table 3:** Score of catalase immunostaining on ICC and normal counterpart tissues

Sample	CAT		SODM		DBI		PRDX6	
Sample	Tumor area	Normal area	Tumor area	Normal area	Tumor area	Normal area	Tumor area	Normal area
1	positive	negative	NA	NA	NA	NA	NA	NA
2	weakly positive	negative	NA	NA	NA	NA	NA	NA
3	positive	negative	NA	NA	NA	NA	NA	NA
4	weakly positive	negative	NA	NA	NA	NA	NA	NA
5	positive	negative	4+	Weak positivity	Strong positivity	Intermediate positivity	negative	negative
6	positive	negative	4+	Weak positivity	Intermediate positivity	Weak positivity	2+	negative
7	positive	negative	3+	Weak positivity	Intermediate positivity	Weak positivity	2+	negative
8	strongly positive	negative	NA	NA	NA	NA	NA	NA
9	positive	negative	3+	Weak positivity	Strong positivity	Intermediate positivity	3+	Weak positivity
10	positive	negative	4+	Intermediate positivity	Intermediate positivity	Weak positivity	2+	negative
11	positive	negative	4+	negative	Weak positivity	Weak positivity	4+	Weak positivity
12	positive	negative	4+	negative	Weak positivity	Weak positivity	negative	negative
13	strongly positive	negative	3+	Weak positivity	Intermediate positivity	Weak positivity	2+	negative
14	positive	negative	4+	Intermediate positivity	Intermediate positivity	Intermediate positivity	negative	negative
15	positive	negative	4+	Weak positivity	Strong positivity	Intermediate positivity	3+	Weak positivity
16	NA	NA	4+	Weak positivity	Strong positivity	Weak positivity	2+	negative

*NA* not available

CAT was detected in all tumor tissue samples with different staining score: 2 out of 15 (13.3%) were weakly positive, 11 out of 15 (73.3%) were positive, and 2 out of 15 (13.3%) were strongly positive. SODM is overexpressed in all tumor tissues, 8 out of 11 tumors (73%) are classified as 4+ and 3 out of 11 tumors (27%) as 3+. PRDX6 is expressed in 8 out 11 samples (73%); of them, 5 are classified as 2+, 2 as 3+, one as 4+ and 3 were negative. For these two proteins, the expression was mainly weak or absent in the normal surrounding bile duct. DBI/ACBP was expressed in all the samples with different intensities, 2 with weak, 5 with intermediate, and 4 strong intensities.

**Fig. 2:**
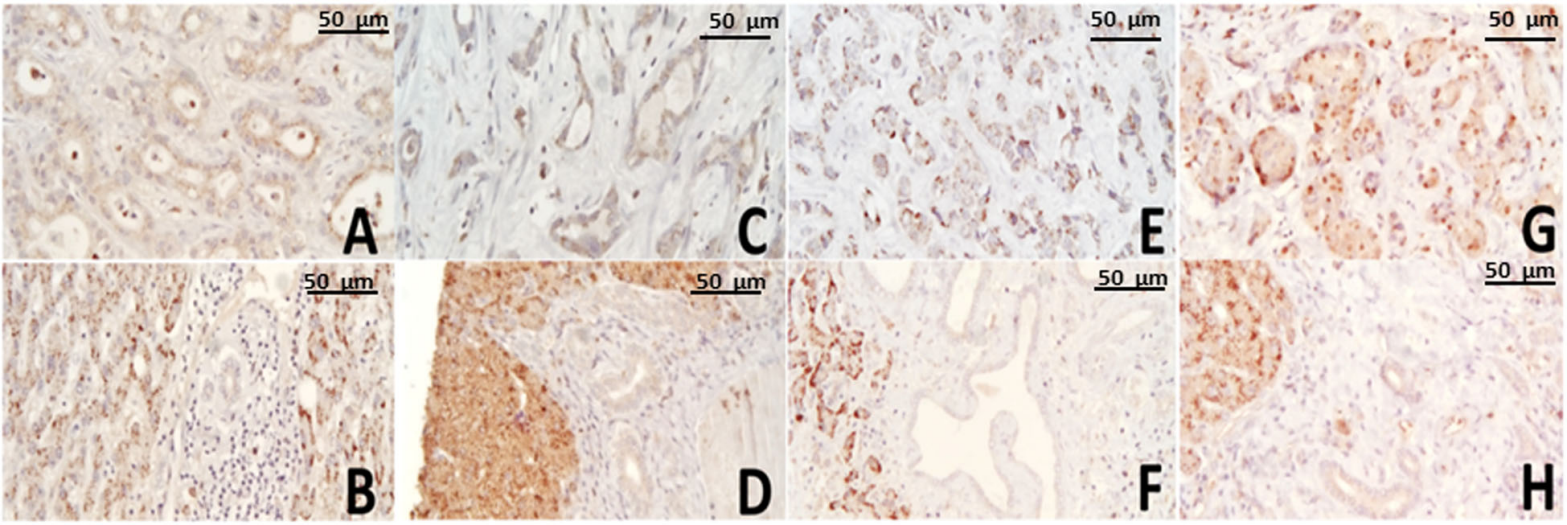
Representative CAT immunostaining images of ICC tissues. A-C-E-G showed ICC tissues with different CAT staining (**A**: weakly positive; **C**: positive; **E**-**G**: highly positive). **B**-**D**-**F**-**H** showed the corresponding normal surrounding tissues. Images were acquired at 40X

In all tested samples, the normal counterpart expressed lower levels of the proteins examined. Figure 2 represents different score staining for CAT in ICC samples, while representative IHC images of tumor sections compared to the normal counterparts for ACBP/DBI, PRDX6, and SODM are shown in Additional file 3.

We exploited the Human Protein Atlas database to retrieve more information about protein expression. CCA is included in the “liver cancer” disease, and for each protein selected, a different number of CCA samples were available. Six out of 8 expressed CAT, one with strong intensity. SODM was highly expressed in 3 out of 4 CCA available, PRDX6 in 6 out of 7 CCAs (four with strong-moderate expression), and for ACBP/DBI only three samples were available, 2 of them with weak expression and one moderate. Additional file 4 summarized data obtained for all the differentially expressed proteins by using Protein Atlas database.

### Comparison between proteomic and transcriptomic data

In a previous work [22], we analyzed the gene expression profiling of 13 ICC (including the five samples used for proteomic analysis) fresh frozen tumors comparing them with a dataset including normal bile ducts (GSE107102). From this dataset, we extrapolated and reanalyzed data of the five samples of interest to investigate if there was a correspondence in terms of expression between protein and transcriptomic data. As shown in Table 4, considering protein expression as reference, and applying a logFC threshold of |0.5| for mRNA expression, we have a protein-mRNA expression concordance of 50%, 5 out of 12 among up- and 4 out of 6 among down-regulated targets in tumors compared to normal tissue.

**Table 4:** Comparison of protein and mRNA expression in ICC samples

Protein ID	Protein Name	Expression in ICC vs normal	Gene Name	Log2FC in ICC vs normal
P07108	ACBP	UP	DBI	0.530
P68871	HBB	UP	HBB	−0.343
P04040	CATA	UP	CAT	−2-284
Q03154	ACY1	UP	ACY1	−0.22
P47985	UCRI	UP	UQCRFS1	0.094
P63261	ACTG	UP	ACTG	−1.213
P30041	PRDX6	UP	PRDX6	− 0993
P32119	PRDX2	UP	PRDX2	0.747
Q96IU4	ABHEB	UP	CIB1	1.103
P04179	SODM	UP	SOD2	1.968
P62805	H4	UP	HIST1H4A	2.571
P00352	ALIA1	UP	ALDH1A1	−3.260
P54868	HMCS2	DOWN	HMGCS2	− 1809
P07237	PDIA1	DOWN	P4HB	0.018
O95954	FTCD	DOWN	FTCD	−3.798
P38646	GRP75	DOWN	HSPA9	−2.673
P68363	TBA1B	DOWN	TUBA1B	−0.939
P06753	TPM3	DOWN	TPM3	0.936

We exploited GEPIA database to compare the expression of these targets between tumor and normal tissue at mRNA levels; 36 ICC and 9 normal tissues are included in the analysis (TCGA dataset). Boxplot for each targets were summarized in Additional file 5 and the concordance between TCGA data and our GEP analysis and TCGA data and proteomic analysis was reported. Results are only partially comparable, mainly due to the small number of samples analyzed in each case series. No association between targets expression and survival was found using TCGA dataset (Additional file 6), with the exception of PRDX2, whose high expression in associated with poor disease free survival (*p* = 0.01) and of ALDH1A1, whose low expression is associated with poor disease free survival (*p* = 0.03).

## Discussion

In the last decade, the identification of putative targets for CCA treatment has become challenging. To date, mutational and transcriptomic profiles, as well as methylation status and fusions assessment, are deeply investigated and many progresses in terms of classification and identification of prognostic biomarkers have been made. It is well-known that all the above-mentioned alterations are strictly associated to ethnicity and etiology and we assume the same behavior for proteomic profile. This, together with the increasing incidence of ICC in Italy and the lack of effective therapies prompted us to analyze the protein expression profile of a small cohort of ICC derived from Italian patients. The first limit of this study is the small number of patients available, but it can be considered a training analysis, which suggests potential targets suitable for therapies to be tested in a validation set. Nevertheless, this study pointed out the impairment of the antioxidant system, with a subsequent accumulation of free radicals. In particular, the main process in which the deregulated proteins are involved is the redox pathway.

It is well established that metabolic processes play a key role in tumor progression. Here, we evidenced an up-regulation of ACBP1, confirmed also in the independent case series by IHC, which is already described in other tumor types, especially in glioblastoma and astrocytoma [23, 24]. In physiological conditions, its role is the maintenance of lipid metabolism, steroidogenesis, and peptide hormone release; when overexpressed, it supports tumor growth by controlling the availability of long chain fatty acids which are processed by mitochondria with a fatty oxidation reaction [25]. ACBP1/DBI silencing induces cell senescence, reduces cell proliferation, delays tumor initiation and prolongs survival in in vivo model. Moreover, due to its role as adaptor to microenvironment changes, it seems to promote the cancer stem cell niche during neurogenesis [26].

HBB is involved in oxygen metabolism. High expression was detected in breast cancer, in particular in bone metastasis [27]. Authors suggested a positive correlation between HBB expression and ability of disseminating tumor cells in other organs, indicating a more aggressive phenotype [28]. This data is also confirmed by the work of Zheng and coll. in which HBB is abundantly expressed in circulating tumor cells of breast and prostate cancer patients and its expression is closely detected in circulating tumor cells (CTC), and not in primary tumors [29]. CAT and SODM, both involved in antioxidant processes, are up-regulated in our cohort of ICC patients. Loilome and collaborators demonstrated, in an *O. viverrini* hamster cholangiocarcinoma model, that both enzymes are highly expressed during cholangiocarcinogenesis, while there is a decreased expression when tumors are well established [30]. The same group demonstrated that both proteins are expressed at different levels in CCA tissues, but they are also expressed in normal bile ducts and hepatocytes [31]. Interestingly, its activity is dramatically reduced in CCA compared to normal bile ducts. In contrast, high expression and activity of antioxidant enzymes, among them SODM and CAT, are found in a cholangiocytes hydrogen peroxide resistant cell line obtained by gradual and continuous exposition to hydrogen peroxides. This cell line had a higher proliferation rate and a more aggressive phenotype compared to the parental one; thus, it may be a suitable model of cholangiocarcinogenesis [32]. In our validation case series, we found that CAT protein is expressed at different levels in cancer tissues, but not in normal adjacent ones. A recent work demonstrated that the presence of variants in genes associated to oxidative stress pathway may affect the response to chemotherapy. Moreover, CAT overexpression inhibits proliferation in vitro of CCA in vitro models and promotes cisplatin and doxorubicin-induced antitumor activity, while low levels of CAT induce resistance to these chemotherapeutic agents [33]. We analyzed the expression of another protein strictly associated with CAT, SODM, found overexpressed by proteomic data. The same trend was revealed in the independent case series tested by IHC.

ACY1 is found up-regulated in different tumor types. Literature data demonstrated that ACY1 knockdown in colorectal cancer cells inhibits proliferation and increases apoptosis, becoming an interesting target to explore [34]. In contrast, ACY1 is a putative tumor suppressor in small cell lung cancer and hepatocarcinoma [35, 36].

The impairment of UQCRFS1, involved in mitochondrial stability, electron transport driving oxidative phosphorylation, expression was described in gastric cancers where it is frequently amplified and associated to tumor progression [37]. Opposite results are shown in clear cell renal carcinoma; UQCRFS1 is downregulated, probably due to a DNA hypermethylation of that region [38]. ACTB expression is high in tumor tissues and cell lines; its deregulation in tumors is associated to loss of polarization and major invasiveness and metastatic potential [39], also described in metastatic breast cancer [40]. PRDX2 is already described as overexpressed in CCA tissues compared to the normal surrounding ones [40], while PRDX6 is overexpressed in the inflammation process induced by Clonorchis Siniensis [41]. The up-regulation of both PRDX2 and PRDX6 is described in many tumors and correlated with invasiveness, migration, drug resistance and enhancing stem cell properties, in particular in NSCLC, colorectal cancer, and esophageal carcinoma [42–45]. From IHC analysis, we found that about 73% of ICC expressed higher levels of PRDX6 compared to the normal adjacent tissues, in line with published data. PRDX6 overexpression is also associated with poor prognosis and overall survival in ovarian cancer [46]. HIST1H4A resulted highly up-regulated in exosomes released by NSCLC [47]. A recent study conducted on CCA patients showed that high expression of ALDH1A1 correlated with a more favorable prognosis [48]; in many studies, ALDH1A1 is a cancer stem cell marker and a suitable target for therapy and only its activity is associated to worse prognosis [49, 50].

Another limit of this study is the use of normal immortalized colangiocytes cell line as control in MS experiments, instead of the most appropriate normal biliary tissue; in fact, the cell line lacks the microenvironment, the cellular components usually present in tumor surrounding tissues, actually weakening and potentially impairing our findings. However, the proteins identified in our study were validated in an independent cohort of ICC tissues comparing their expression with the normal surrounding tissues, albeit on a limited number of cases.

Globally, this study, even if conducted on a small number of samples, provided precious information about the role of oxidative and metabolic processes in CCA progression, suggesting also that they may be good targets for therapy in CCA. Combining therapies able to tip the balance towards the anti-cancer activity of these pathways with standard chemotherapy could be an alternative approach in CCA treatment. Recently, it was demonstrated that the administration of metformin in association to Cisplatin enhances the oxidative stress mediated cell death pathway, hence increasing the efficacy of Cisplatin alone [50]. Moreover, these pathways may have a potential as prognostic biomarkers in serum. Uchida and collaborators demonstrated that an increase concentration of reactive oxygen metabolites and a decrease level of anti-oxidative metabolites in serum are associated to poor outcome in CCA patients, suggesting the importance of such processes in tumor progression [51]. The complexity of metabolic and oxidative pathways deserves tailored studies to clarify their role in cancer development, progression and drug resistance.

## Supplementary Information

**Additional file 1.** Additional methods. MS spectra data acquisition.

**Additional file 2.** 2D gel images of single ICC samples. The molecular weight on the left side corresponds to (from top to bottom): 250 kDa, 150 kDa, 100 kDa, 75 kDa, 50 kDa, 37 kDa, 25 kDa, 20 kDa, 15 kDa, 10 kDa.

**Additional file 3.** Representative images of IHC for DBI, PRDX6, and SODM. DBI staining in tumor tissue A) in normal counterpart B); PRDX6 staining in tumor tissue C) in normal counterpart D); SODM staining in tumor tissue E) in normal counterpart F). All the images are captured with 20X.

**Additional file 4.** Protein expression data obtained from The Protein Atlas database.

**Additional file 5.** Box plots representing the expression at mRNA level of all the targets identified by proteomics obtained using GEPIA. Red box: ICC; grey box: normal tissues.

**Additional file 6.** Kaplan Meier curves (Overall survival and Disease free survival) obtained using GEPIA exploiting mRNA expression data of TCGA.

## Data Availability

The datasets used and/or analyzed during the current study are available from the corresponding author on reasonable request.

## Abbreviations

CCA: Cholangiocarcinoma
ICC: Intrahepatic cholangiocarcinoma
MS: Mass spectrometry
CAT: Catalase
SOD: Superoxide dismutase
PRDX6: Peroxiredoxin 6
DBI/ACBP: Diazepam Binding Inhibitor, Acyl-CoA Binding Protein
FFPE: Formalin fixed, paraffin-embedded
ECC: Extrahepatic cholangiocarcinoma
iTRAQ: Isobaric tags for relative and absolute quantitation
FF: Fresh frozen
IEF: Isoelectric focusing
DTT: Dithiothreitol
ACN: Acetonitrile
TBS: Tris buffered saline
2-D GE: 2 dimentional gel electrophoresis
FC: Fold change
