# Supplementary material for: Proteomic analysis identifies deregulated metabolic and oxidative-associated proteins in Italian intrahepatic cholangiocarcinoma patients
Source: BMC Cancer. 2021 Jul 28;21:865. doi: 10.1186/s12885-021-08576-z (PMC8317365; doi:10.1186/s12885-021-08576-z)
Supplement: Supplementary file 1 — Additional file 1. Additional methods. MS spectra data acquisition. [file 12885_2021_8576_MOESM1_ESM.docx]

**Additional methods**

**MS spectra data acquisition**

The spectra were acquired in the positive reflector mode by 20 subspectral accumulations (each consisting of 50 laser shots) in an 800–4000 mass range, focus mass 2100 Da, using a 355 nm Nb:YAG laser with a 20 kV acceleration voltage. Peak labeling was automatically done by 4000 Series Explorer software Version 4.1.0 (Sciex) without any kind of smoothing of peaks or baseline, considering only peaks that exceeded a signal-to noise ratio of 10 (local noise window 200 m/z) and a half maximal width of 2.9 bins. Calibration was performed using default calibration originated by five standard spots (Mass Standards kit for Calibration P/N 4333604). Only MS/MS spectra of preselected peaks were integrated over 1000 laser shots in the 1 kV positive ion mode with the metastable suppressor turned on. Air at the medium gas pressure setting (1.25 × 10^−6^ Torr) was used as the collision gas in the CID off mode. After smoothing and baseline subtractions, spectra were generated automatically by 4000 Series Explorer software. MS and MS/MS spectra were processed by ProteinPilot Software 4.5 (Sciex) which acts as an interface between the Oracle database containing raw spectra and a local copy of the MASCOT search engine (Version 2.1, Matrix Science, Ltd.). The Paragon algorithm of ProteinPilot Software 4.5 was used with identification as the Sample Type, iodacetamide as cysteine alkylation, with the search option “biological modifications” checked, and trypsin as the selected enzyme (one missed cleavage site was allowed). MS/MS protein identification was performed against the human Uniprot/SwissProt database using a confidence threshold of 95% (Proteinpilot Unused score ≥ 1.31). The monoisotopic precursor ion tolerance was set to 0.12 Da and the MS/MS ion tolerance to 0.3 Da. Two peptides were required for protein identification.
