# Supplementary material for: Proteomic analysis identifies deregulated metabolic and oxidative-associated proteins in Italian intrahepatic cholangiocarcinoma patients
Source: BMC Cancer. 2021 Jul 28;21:865. doi: 10.1186/s12885-021-08576-z (PMC8317365; doi:10.1186/s12885-021-08576-z)
Supplement: Supplementary file 2 — Additional file 2. 2D gel images of single ICC samples. The molecular weight on the left side corresponds to (from top to bottom): 250 kDa, 150 kDa, 100 kDa, 75 kDa, 50 kDa, 37 kDa, 25 kDa, 20 kDa, 15 kDa, 10 kDa. [file 12885_2021_8576_MOESM2_ESM.docx]

**Additional file 2. Pictures of the gels of the other four ICC samples.**

The molecular weight on the left side corresponds to (from top to bottom): 250 kDa, 150 kDa, 100 kDa, 75 kDa, 50 kDa, 37 kDa, 25 kDa, 20 kDa, 15 kDa, 10 kDa.


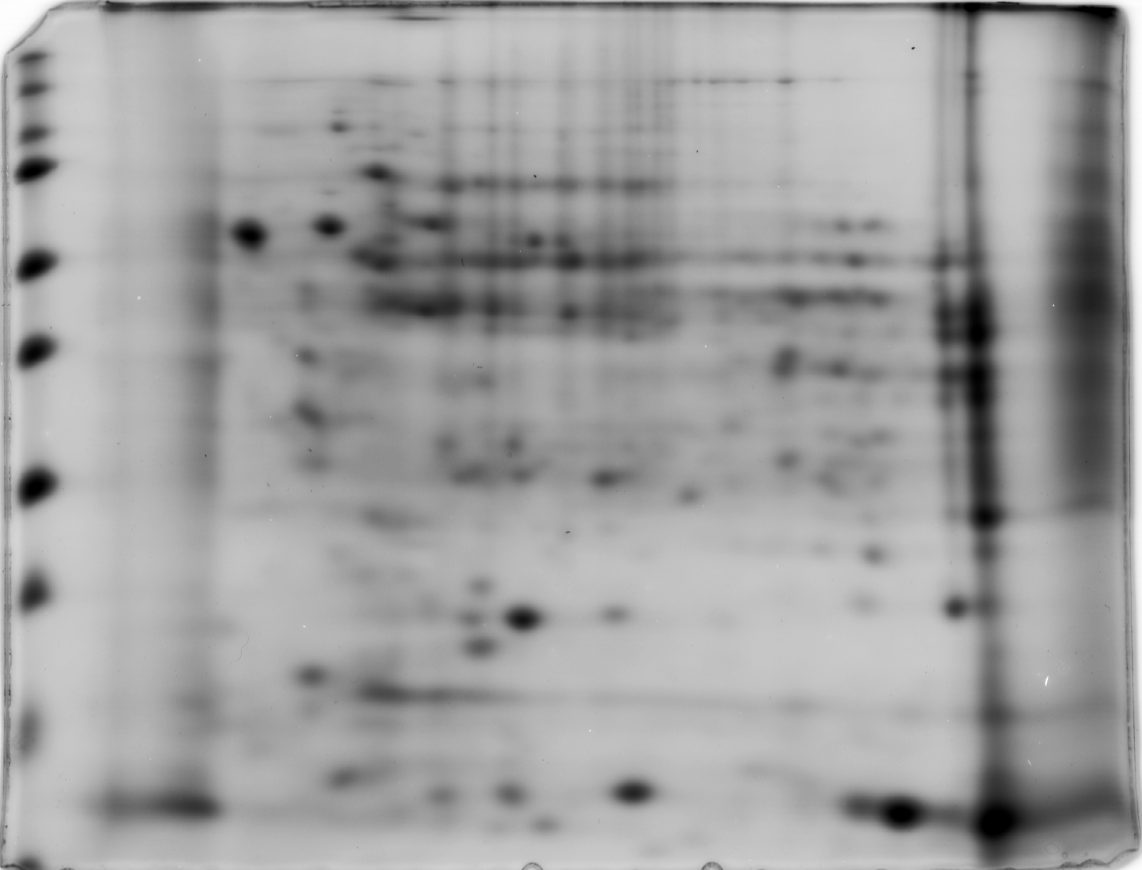


**Figure e1. Gel image of Sample 2**


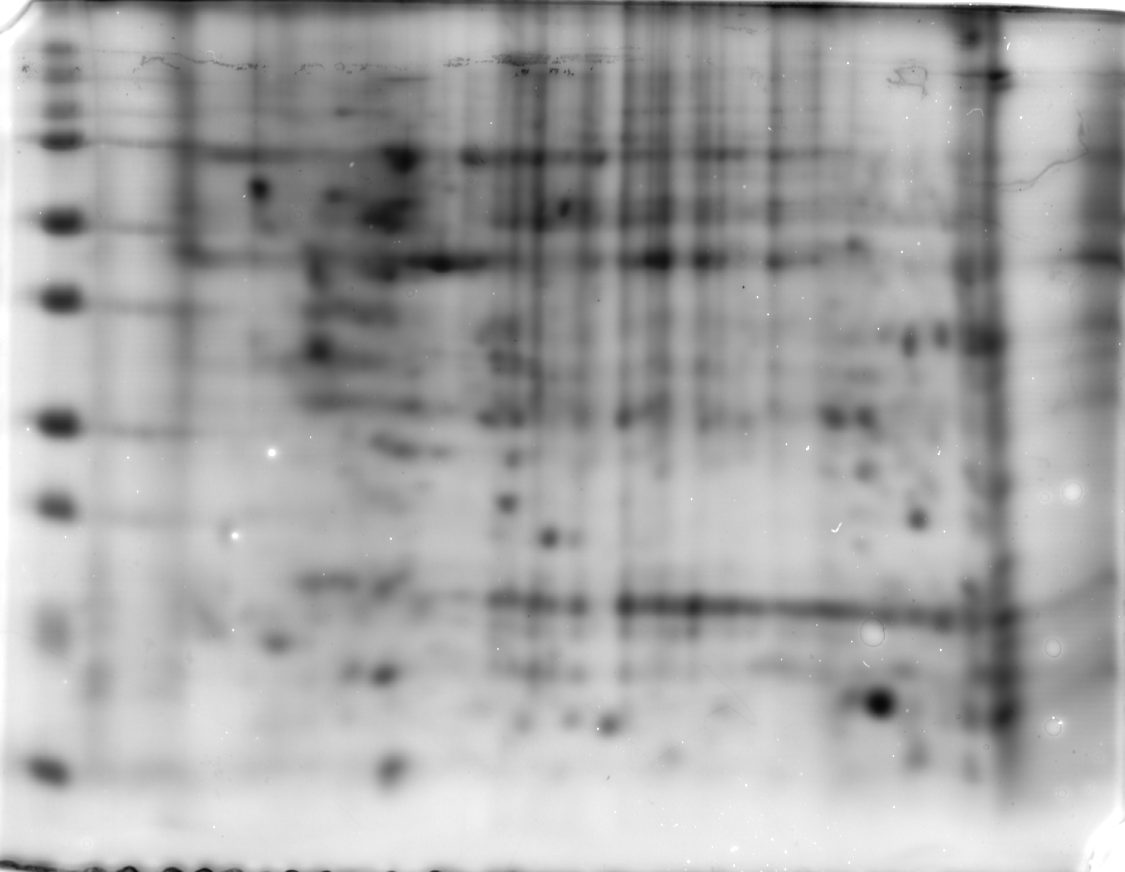


**Figure e2. Gel image of Sample 3**

**
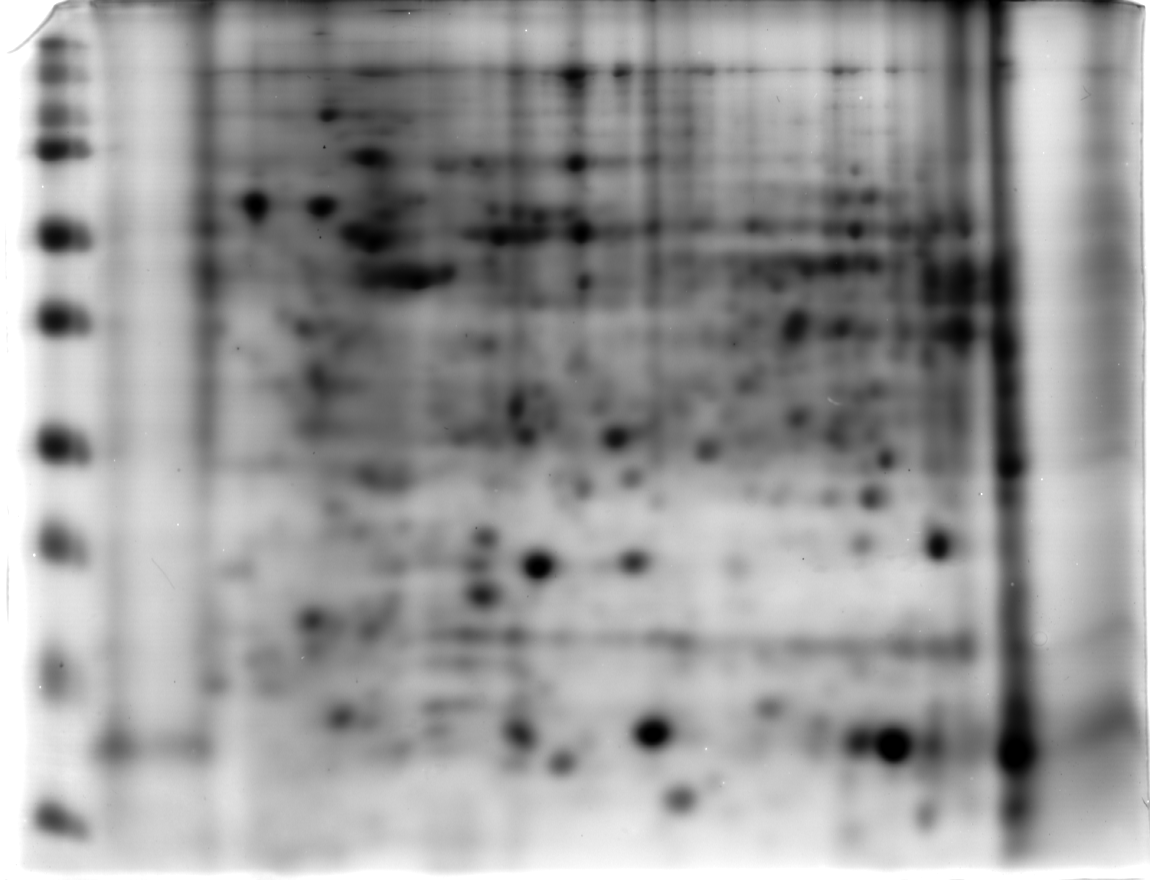
**

**Figure e3. Gel image of Sample 4**

**
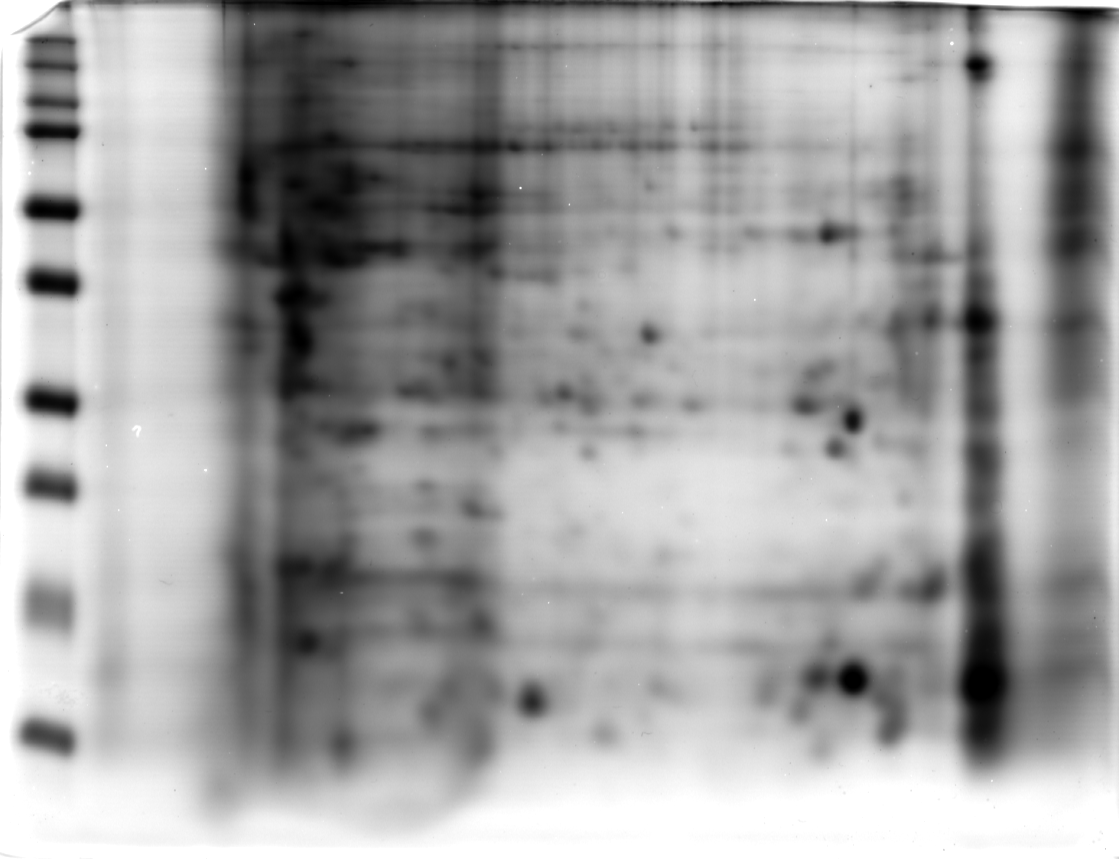
**

**Figure e4. Gel image of Sample 5**
