# Supplementary figures and images for: Proteomic analysis identifies deregulated metabolic and oxidative-associated proteins in Italian intrahepatic cholangiocarcinoma patients
Source: BMC Cancer. 2021 Jul 28;21:865. doi: 10.1186/s12885-021-08576-z (PMC8317365; doi:10.1186/s12885-021-08576-z)

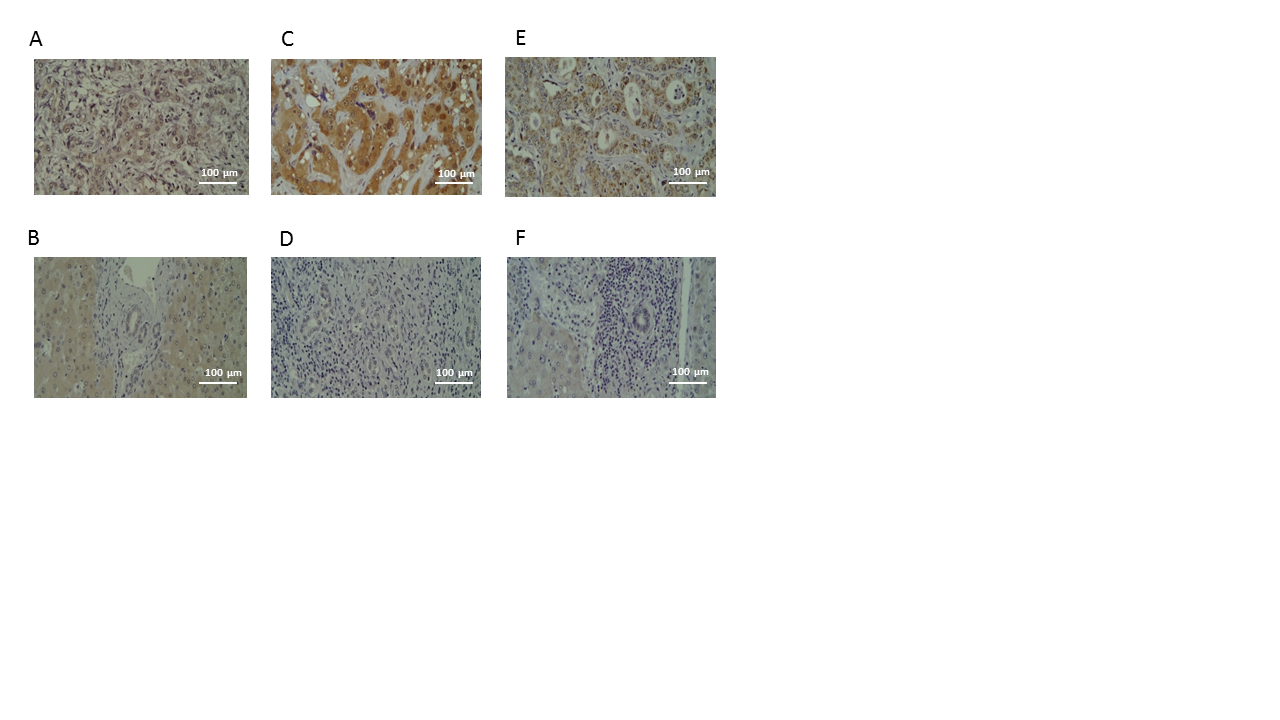

Supplement: Supplementary file 3 — Additional file 3. Representative images of IHC for DBI, PRDX6, and SODM. DBI staining in tumor tissue A) in normal counterpart B); PRDX6 staining in tumor tissue C) in normal counterpart D); SODM staining in tumor tissue E) in normal counterpart F). All the images are captured with 20X. [file 12885_2021_8576_MOESM3_ESM.tif]
