# Supplementary material for: Proteomic analysis identifies deregulated metabolic and oxidative-associated proteins in Italian intrahepatic cholangiocarcinoma patients
Source: BMC Cancer. 2021 Jul 28;21:865. doi: 10.1186/s12885-021-08576-z (PMC8317365; doi:10.1186/s12885-021-08576-z)
Supplement: Supplementary file 5 — Additional file 5. Box plots representing the expression at mRNA level of all the targets identified by proteomics obtained using GEPIA. Red box: ICC; grey box: normal tissues. [file 12885_2021_8576_MOESM5_ESM.pdf]

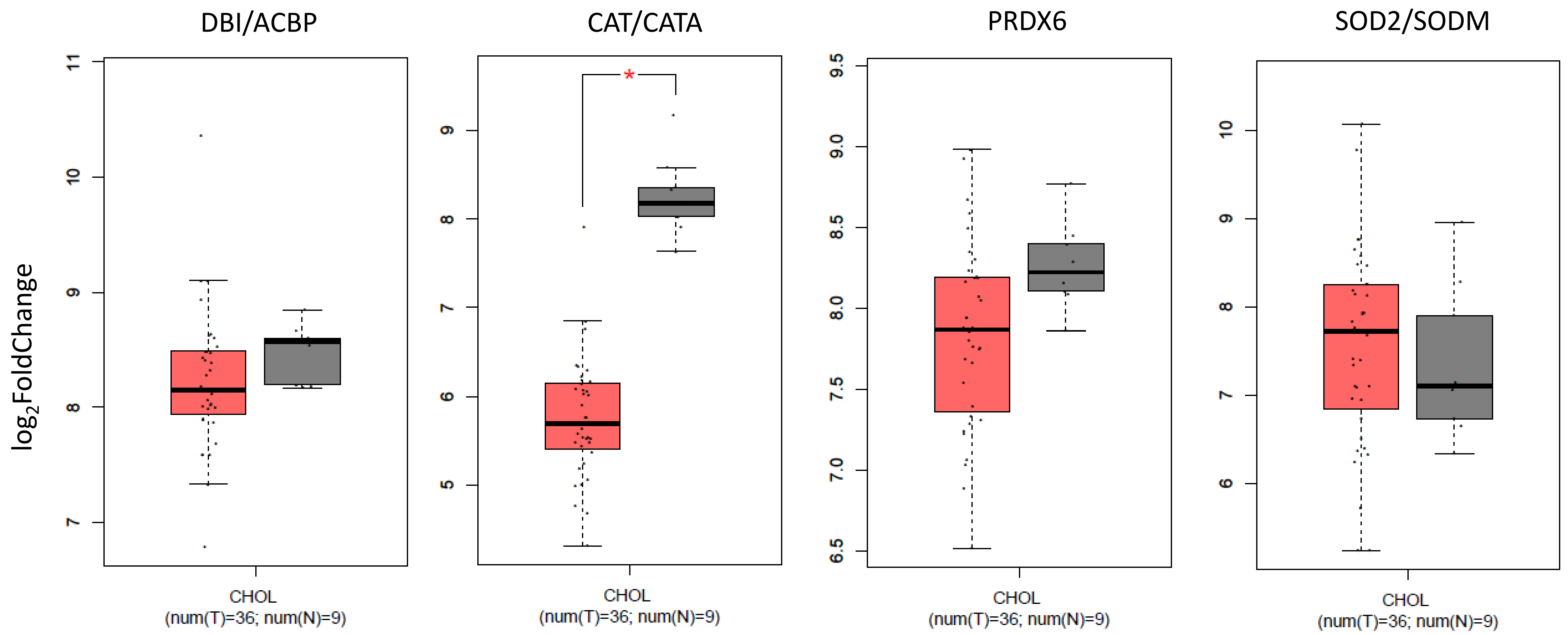

	DBI/ACBP	CAT/CATA	PRDX6	SOD2/SODM
Proteomic data:	discordant	discordant	discordant	concordant
GEP analysis:	discordant	concordant	concordant	concordant

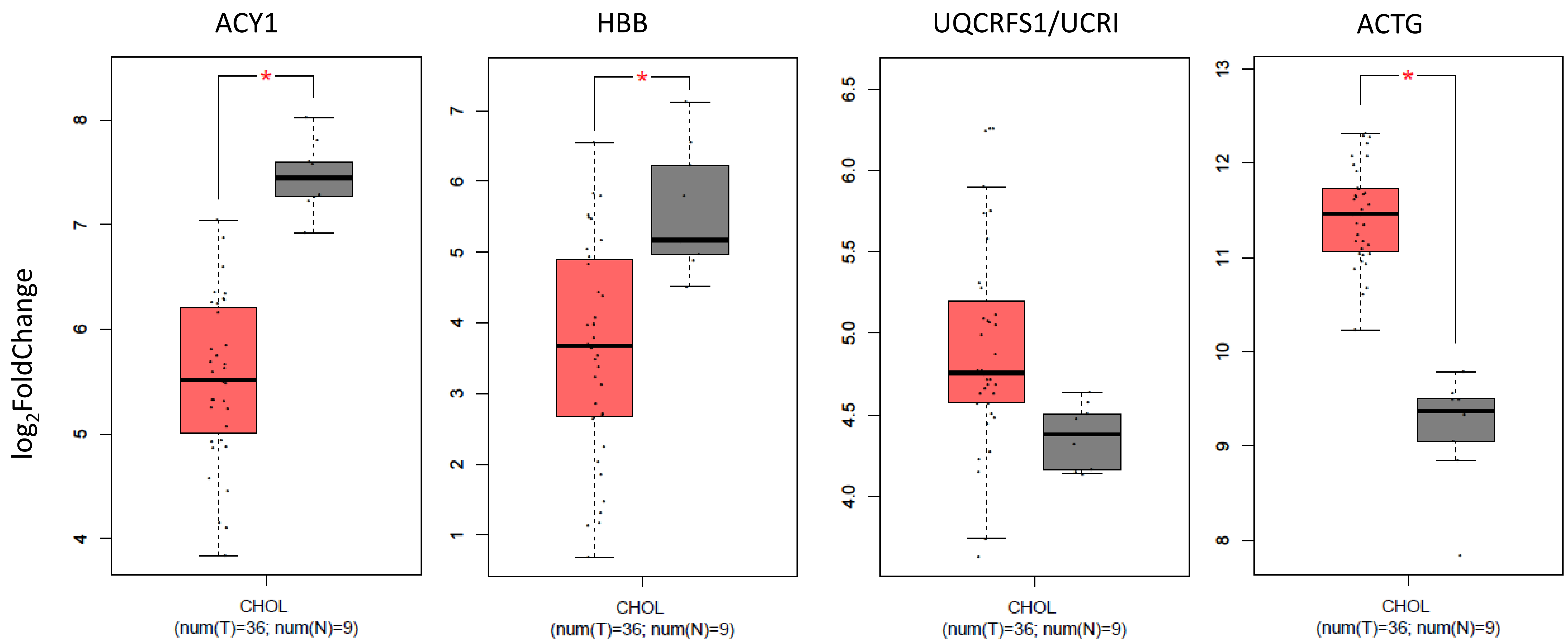

	ACY1	HBB	UQCRFS1/UCRI	ACTG
Proteomic data:	discordant	discordant	concordant	concordant
GEP analysis:	discordant	concordant	discordant	discordant

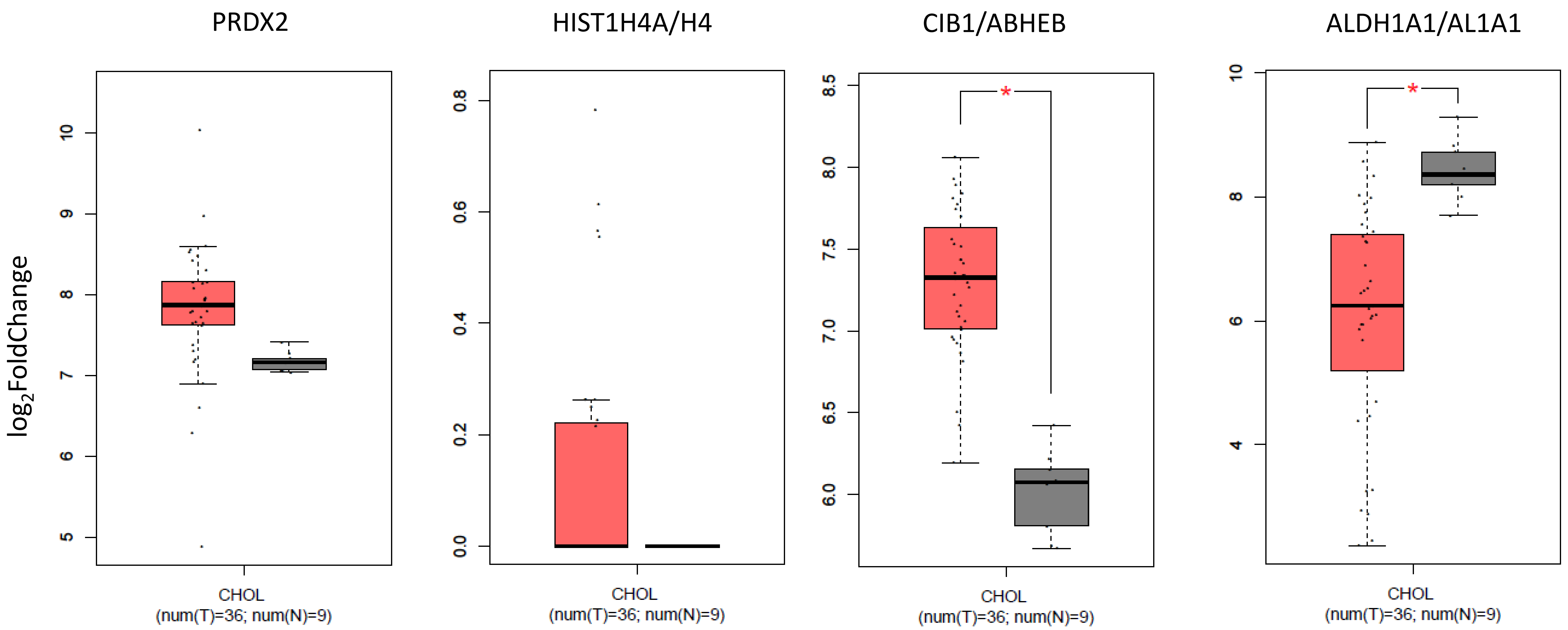

	PRDX2	HIST1H4A/H4	CIB1/ABHEB	ALDH1A1/AL1A1
Proteomic data:	concordant	concordant	concordant	discordant
GEP analysis:	concordant	concordant	concordant	concordant

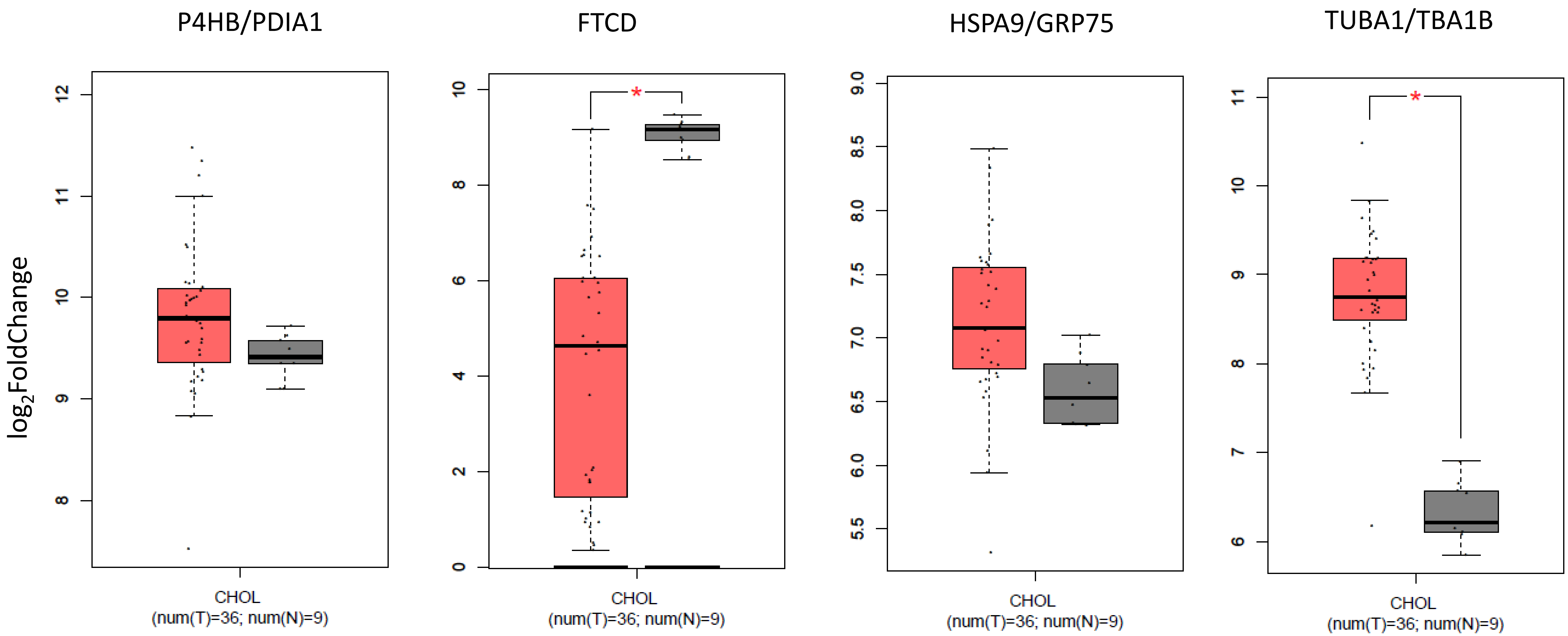

Proteomic data:	discordant	concordant	discordant	discordant
GEP analysis:	discordant	concordant	discordant	discordant

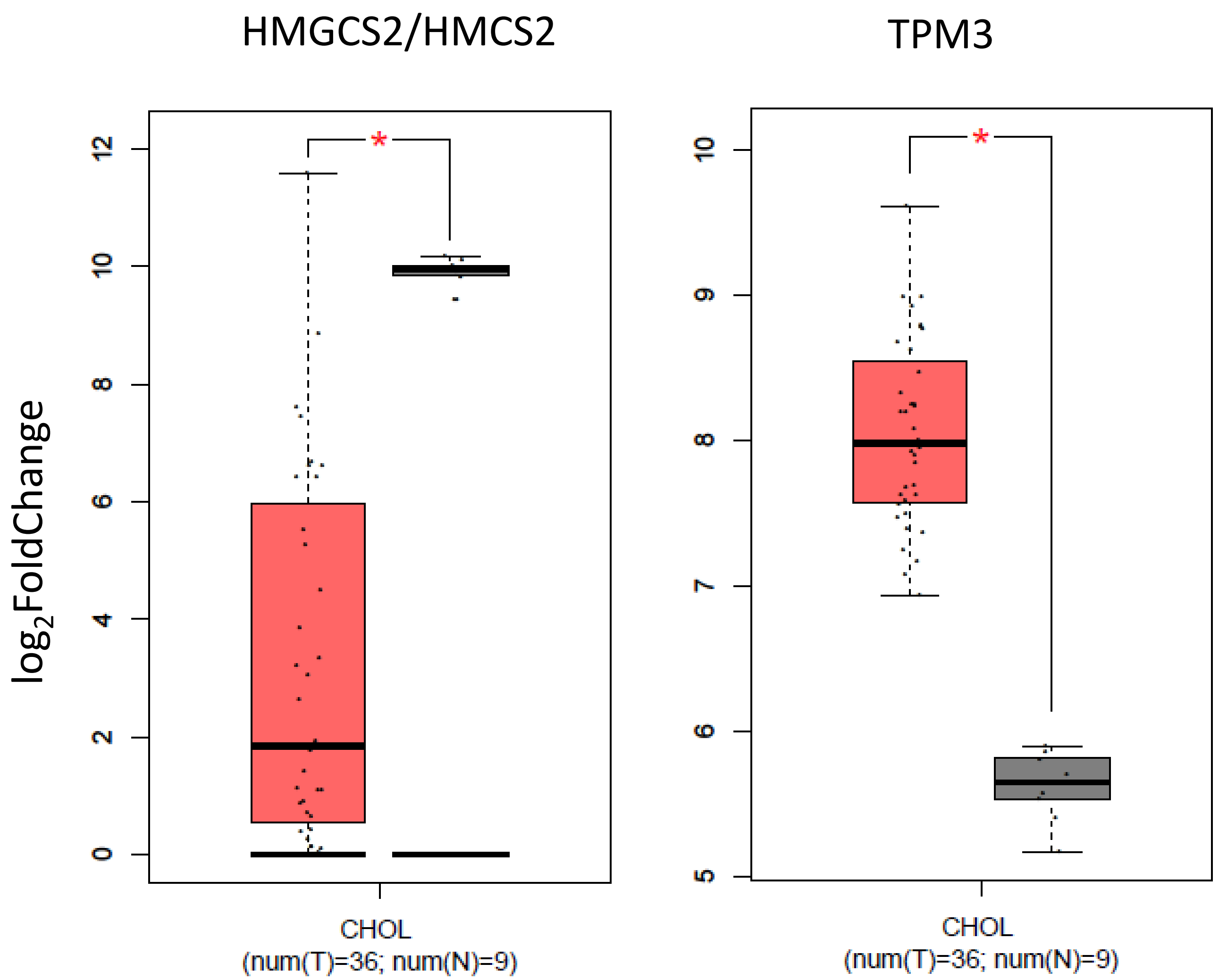

Proteomic data:	concordant	discordant
GEP analysis:	concordant	discordant
