# Supplementary material for: Proteomic analysis identifies deregulated metabolic and oxidative-associated proteins in Italian intrahepatic cholangiocarcinoma patients
Source: BMC Cancer. 2021 Jul 28;21:865. doi: 10.1186/s12885-021-08576-z (PMC8317365; doi:10.1186/s12885-021-08576-z)
Supplement: Supplementary file 6 — Additional file 6. Kaplan Meier curves (Overall survival and Disease free survival) obtained using GEPIA exploiting mRNA expression data of TCGA. [file 12885_2021_8576_MOESM6_ESM.pdf]

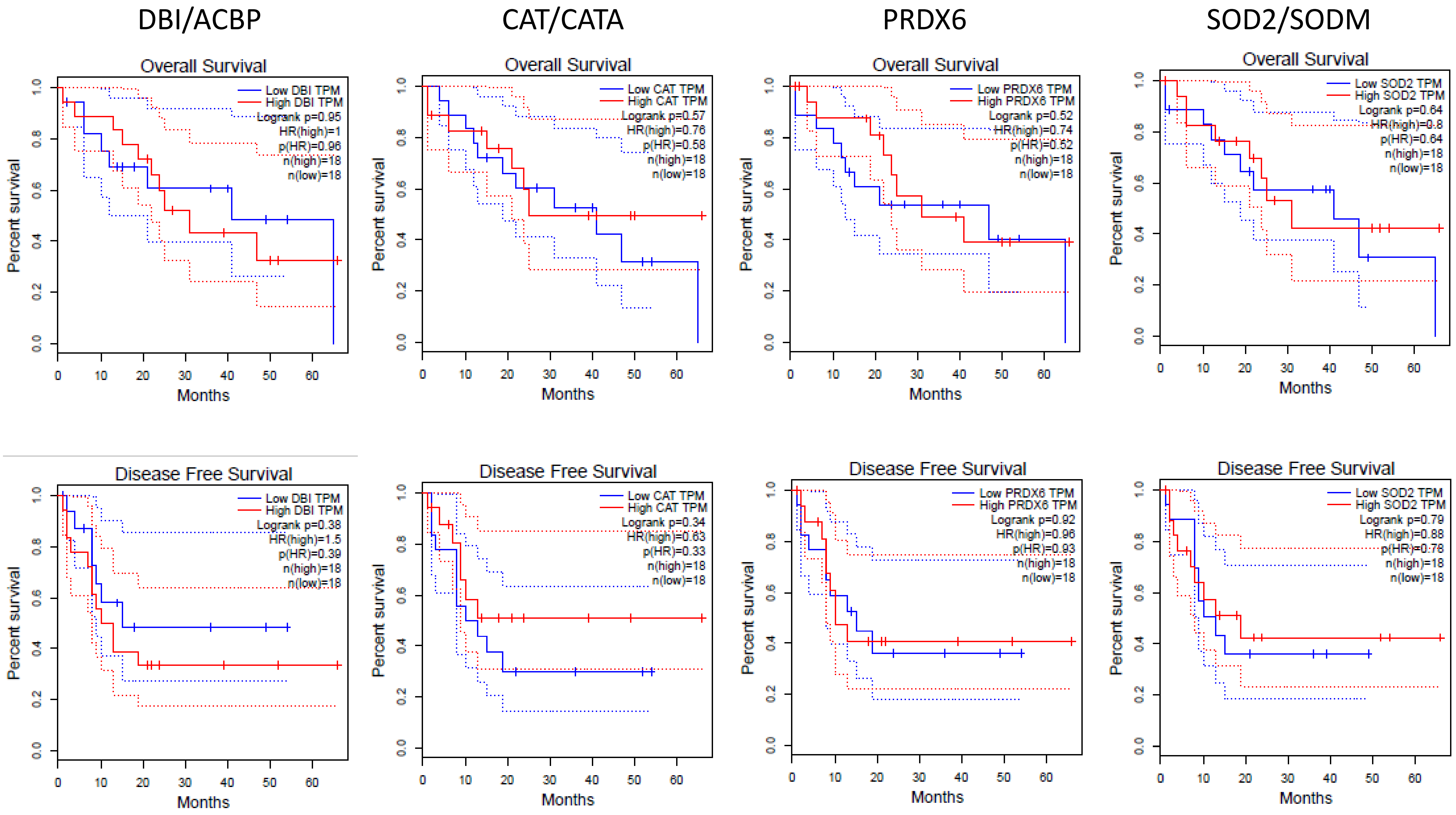
DBI/ACBP
CAT/CATA
PRDX6
SOD2/SODM
Overall Survival
Low DBI TPM
High DBI TPM
Logrank p=0.95
HR(high)=1
p(HR)=0.96
n(high)=18
n(low)=18
Overall Survival
Low CAT TPM
High CAT TPM
Logrank p=0.57
HR(high)=0.76
p(HR)=0.58
n(high)=18
n(low)=18
Overall Survival
Low PRDX6 TPM
High PRDX6 TPM
Logrank p=0.52
HR(high)=0.74
p(HR)=0.52
n(high)=18
n(low)=18
Overall Survival
Low SOD2 TPM
High SOD2 TPM
Logrank p=0.64
HR(high)=0.8
p(HR)=0.64
n(high)=18
n(low)=18
Disease Free Survival
Low DBI TPM
High DBI TPM
Logrank p=0.38
HR(high)=1.5
p(HR)=0.39
n(high)=18
n(low)=18
Disease Free Survival
Low CAT TPM
High CAT TPM
Logrank p=0.34
HR(high)=0.63
p(HR)=0.33
n(high)=18
n(low)=18
Disease Free Survival
Low PRDX6 TPM
High PRDX6 TPM
Logrank p=0.92
HR(high)=0.96
p(HR)=0.93
n(high)=18
n(low)=18
Disease Free Survival
Low SOD2 TPM
High SOD2 TPM
Logrank p=0.79
HR(high)=0.88
p(HR)=0.78
n(high)=18
n(low)=18
Percent survival
Months

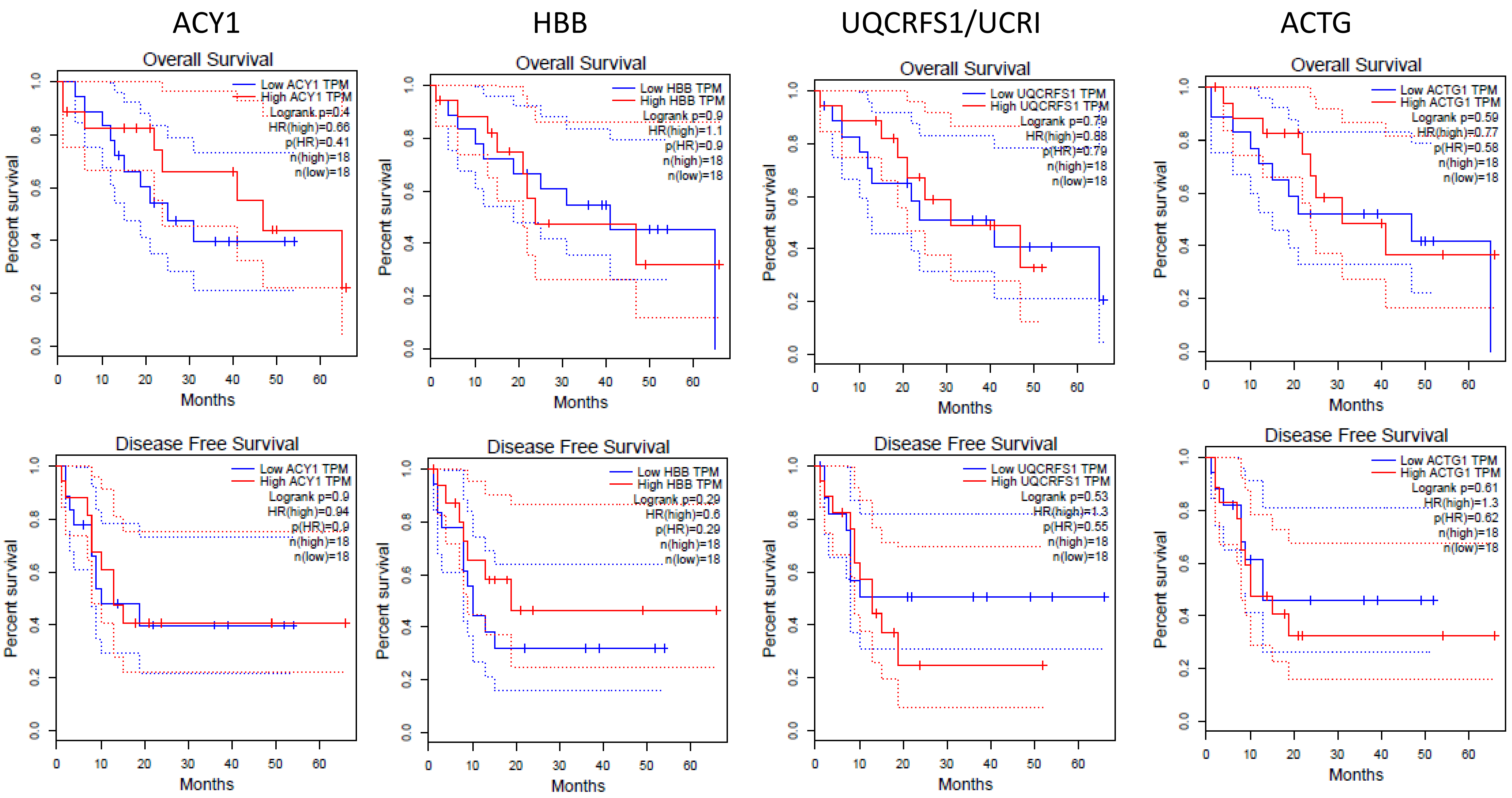
ACY1
HBB
UQCRFS1/UCRI
ACTG
Overall Survival
Low ACY1 TPM
High ACY1 TPM
Logrank p=0.4
HR(high)=0.66
p(HR)=0.41
n(high)=18
n(low)=18
Overall Survival
Low HBB TPM
High HBB TPM
Logrank p=0.9
HR(high)=1.1
p(HR)=0.9
n(high)=18
n(low)=18
Overall Survival
Low UQCRFS1 TPM
High UQCRFS1 TPM
Logrank p=0.79
HR(high)=0.88
p(HR)=0.79
n(high)=18
n(low)=18
Overall Survival
Low ACTG1 TPM
High ACTG1 TPM
Logrank p=0.59
HR(high)=0.77
p(HR)=0.58
n(high)=18
n(low)=18
Disease Free Survival
Low ACY1 TPM
High ACY1 TPM
Logrank p=0.9
HR(high)=0.94
p(HR)=0.9
n(high)=18
n(low)=18
Disease Free Survival
Low HBB TPM
High HBB TPM
Logrank p=0.29
HR(high)=0.6
p(HR)=0.29
n(high)=18
n(low)=18
Disease Free Survival
Low UQCRFS1 TPM
High UQCRFS1 TPM
Logrank p=0.53
HR(high)=1.3
p(HR)=0.55
n(high)=18
n(low)=18
Disease Free Survival
Low ACTG1 TPM
High ACTG1 TPM
Logrank p=0.61
HR(high)=1.3
p(HR)=0.62
n(high)=18
n(low)=18
Percent survival
Months

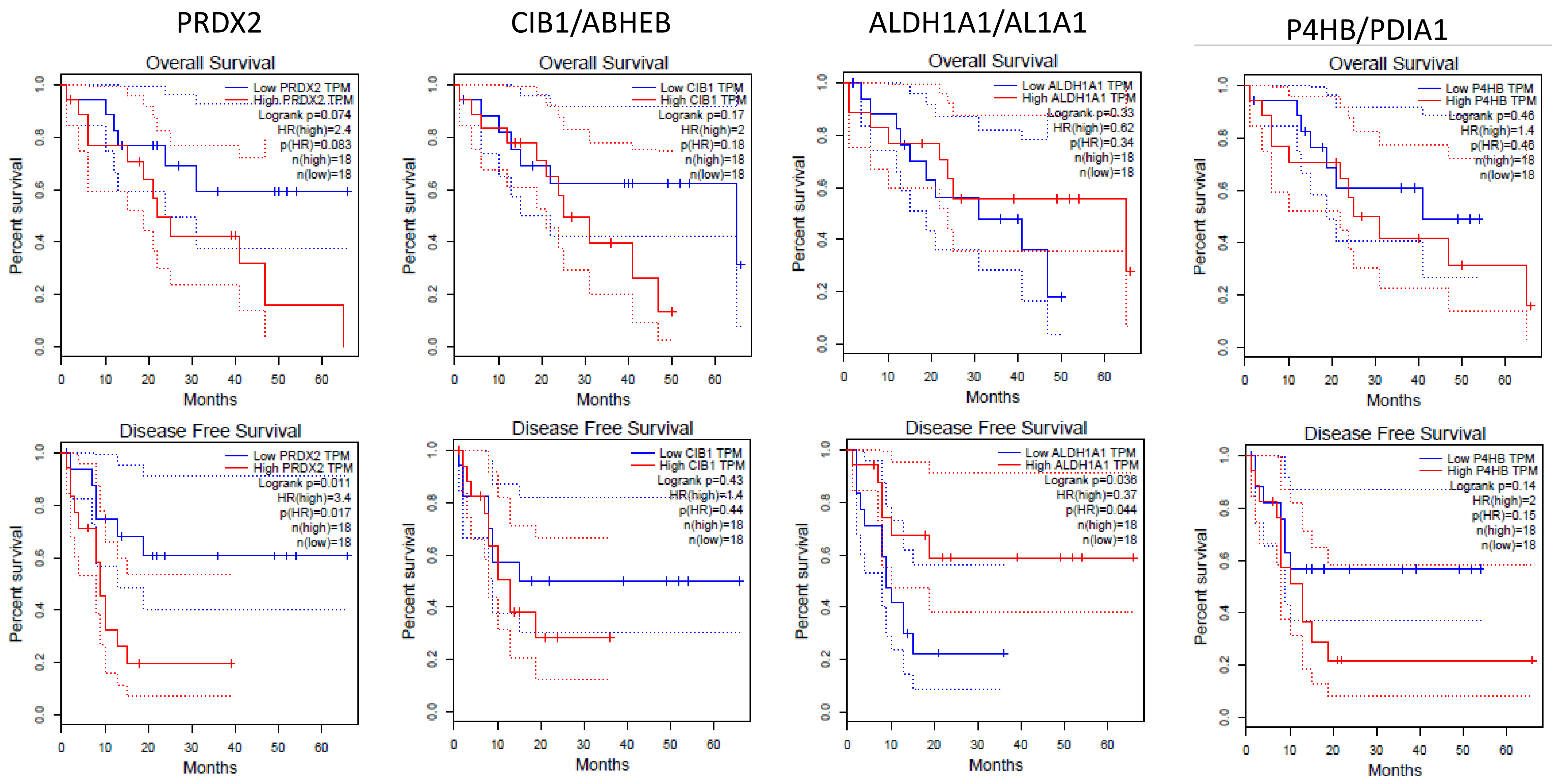
PRDX2
CIB1/ABHEB
ALDH1A1/AL1A1
P4HB/PDIA1
Overall Survival
Low PRDX2 TPM
High PRDX2 TPM
Logrank p=0.074
HR(high)=2.4
p(HR)=0.083
n(high)=18
n(low)=18
Overall Survival
Low CIB1 TPM
High CIB1 TPM
Logrank p=0.17
HR(high)=2
p(HR)=0.18
n(high)=18
n(low)=18
Overall Survival
Low ALDH1A1 TPM
High ALDH1A1 TPM
Logrank p=0.33
HR(high)=0.62
p(HR)=0.34
n(high)=18
n(low)=18
Overall Survival
Low P4HB TPM
High P4HB TPM
Logrank p=0.46
HR(high)=1.4
p(HR)=0.46
n(high)=18
n(low)=18
Disease Free Survival
Low PRDX2 TPM
High PRDX2 TPM
Logrank p=0.011
HR(high)=3.4
p(HR)=0.017
n(high)=18
n(low)=18
Disease Free Survival
Low CIB1 TPM
High CIB1 TPM
Logrank p=0.43
HR(high)=1.4
p(HR)=0.44
n(high)=18
n(low)=18
Disease Free Survival
Low ALDH1A1 TPM
High ALDH1A1 TPM
Logrank p=0.036
HR(high)=0.37
p(HR)=0.044
n(high)=18
n(low)=18
Disease Free Survival
Low P4HB TPM
High P4HB TPM
Logrank p=0.14
HR(high)=2
p(HR)=0.15
n(high)=18
n(low)=18
Percent survival
Months

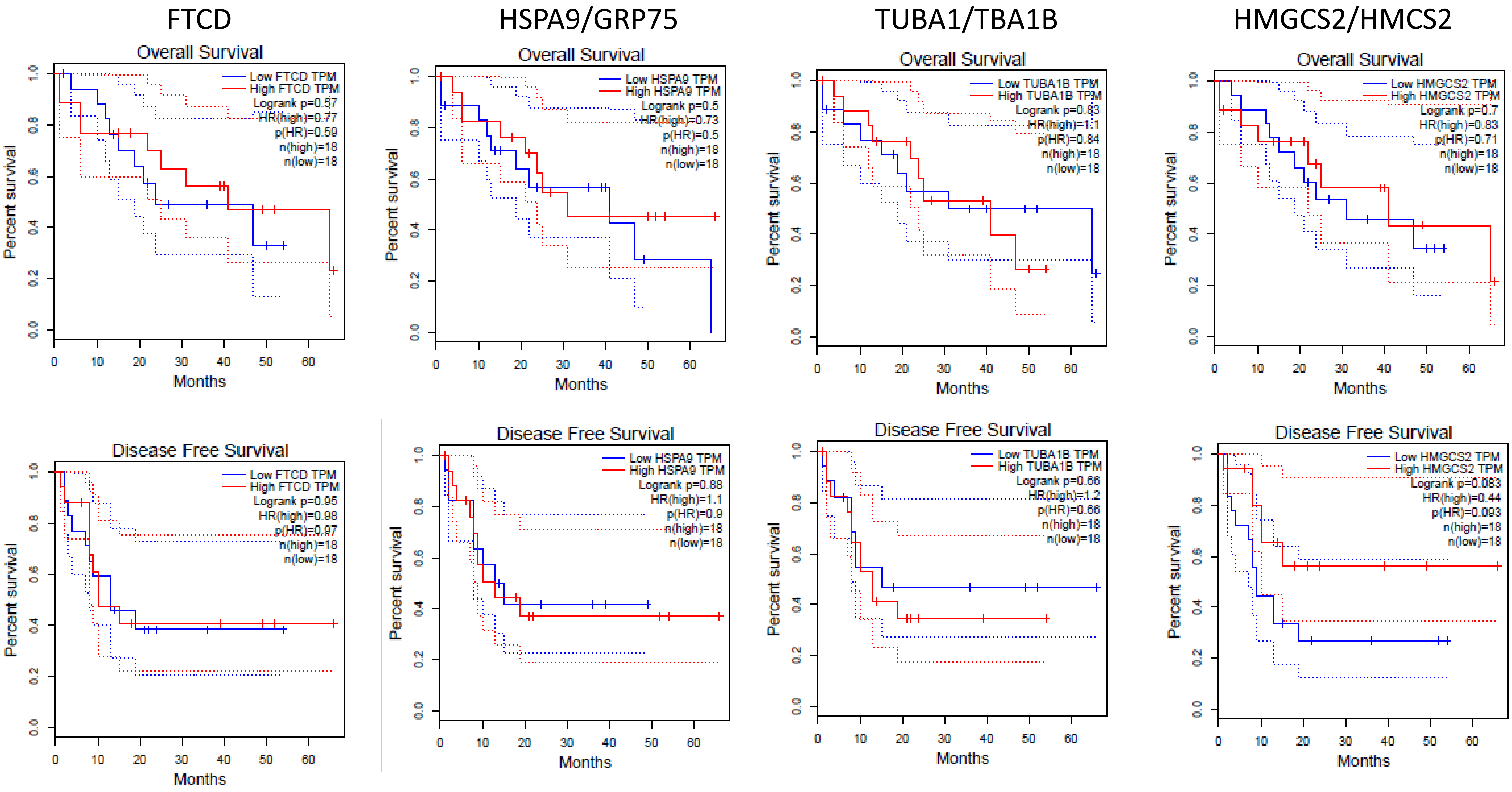
FTCD
HSPA9/GRP75
TUBA1/TBA1B
HMGCS2/HMCS2
Overall Survival
Low FTCD TPM
High FTCD TPM
Logrank p=0.57
HR(high)=0.77
p(HR)=0.59
n(high)=18
n(low)=18
Percent survival
Months
Overall Survival
Low HSPA9 TPM
High HSPA9 TPM
Logrank p=0.5
HR(high)=0.73
p(HR)=0.5
n(high)=18
n(low)=18
Overall Survival
Low TUBA1B TPM
High TUBA1B TPM
Logrank p=0.83
HR(high)=1.1
p(HR)=0.84
n(high)=18
n(low)=18
Overall Survival
Low HMGCS2 TPM
High HMGCS2 TPM
Logrank p=0.7
HR(high)=0.83
p(HR)=0.71
n(high)=18
n(low)=18
Disease Free Survival
Low FTCD TPM
High FTCD TPM
Logrank p=0.95
HR(high)=0.98
p(HR)=0.97
n(high)=18
n(low)=18
Disease Free Survival
Low HSPA9 TPM
High HSPA9 TPM
Logrank p=0.88
HR(high)=1.1
p(HR)=0.9
n(high)=18
n(low)=18
Disease Free Survival
Low TUBA1B TPM
High TUBA1B TPM
Logrank p=0.66
HR(high)=1.2
p(HR)=0.66
n(high)=18
n(low)=18
Disease Free Survival
Low HMGCS2 TPM
High HMGCS2 TPM
Logrank p=0.083
HR(high)=0.44
p(HR)=0.093
n(high)=18
n(low)=18

TPM3

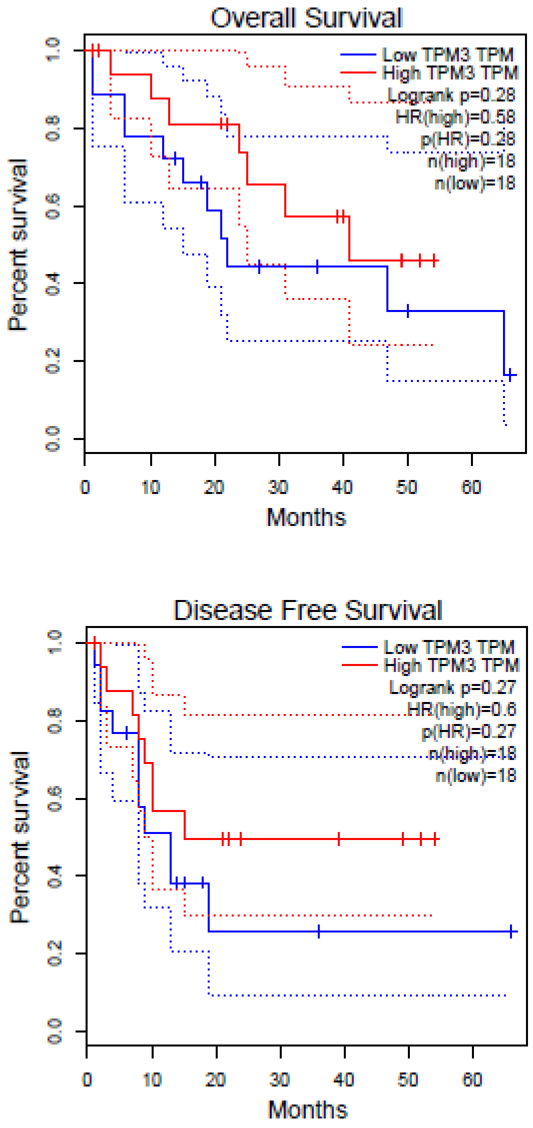
Overall Survival
Low TPM3 TPM
High TPM3 TPM
Logrank p=0.28
HR(high)=0.58
p(HR)=0.28
n(high)=18
n(low)=18
Percent survival
Months
Disease Free Survival
Low TPM3 TPM
High TPM3 TPM
Logrank p=0.27
HR(high)=0.6
p(HR)=0.27
n(high)=18
n(low)=18
Percent survival
Months
